# Influence of medical humanization on patients’ attribution in negative medical situations with communication as the mediator: a questionnaire study

**DOI:** 10.3389/fpubh.2023.1152381

**Published:** 2023-08-31

**Authors:** Peijuan Wang, Yao Wang, Qing Wu, Fan Su, Xin Chang

**Affiliations:** ^1^School of Foreign Languages, Tongji University, Shanghai, Shanghai Municipality, China; ^2^Faculty of Education, East China Normal University, Shanghai, Shanghai Municipality, China

**Keywords:** medical humanization, communication, patients’ attribution in negative medical situations, doctor–patient conflicts, doctor–patient relationships

## Abstract

**Background:**

Patients’ attribution in negative medical situations plays a vital role in reducing medical conflicts and developing high-quality healthcare. The purpose of this study was to investigate the triadic relations among patients’ attribution, medical humanization and communication. Furthermore, the mediating effect of communication was tested.

**Methods:**

A cross-sectional study on the relationship between patients’ attribution in negative medical situations and medical staff’s humanization and communication was conducted, with 3,000 participants totally from 103 hospitals of three different levels in different regions.

**Results:**

There were significant positive correlations among medical staff’s humanization, communication and patients’ attributional styles (*r* = 0.112–0.236, *p* < 0.001 for all). Medical humanization had direct predictive effects on patients’ attributional style in negative medical situations (*β* = 0.14, *p* < 0.01). Mediation analysis also indicated the indirect predictive effect of medical humanization on patients’ attributions through communication (*β* = 0.02, *p* < 0.01).

**Conclusion:**

Patients’ attribution in negative medical situations is predicted by patients’ perception of medical staff’s humanization in healthcare and physicians’ communication skills. Medical humanization not only affects patients’ attributions in negative situations directly, but also influences patients’ attributions via communication indirectly. The humanistic care should be included in medical education for healthcare professionals, and professional training on medical staff’s humanization and communication skills is strongly needed to establish healthy and harmonious doctor–patient relationship.

## 1. Background

Doctor–patient conflicts and even medical disputes occur frequently in contemporary Chinese society, resulting in a variety of tragedies. For example, in 2019, a family member of a patient was unsatisfied with the doctor’s treatment for his mother, thus he killed the doctor in the emergency rescue room (1). In (2), a patient stabbed a doctor in the clinic in a hospital of Shenzhen because he attributed his poor medical outcome to the doctor instead of himself even though the unsatisfactory medical effect was actually due to himself (2). In fact, whether patients engage in aggressive behavior towards doctors is inseparable from patients’ attribution and their perception of others’ intentions (3–6). For example, a study of patient hostility to health care professionals found that the fear that patients often experience impairs cognitive processing, making it difficult to make accurate attributions (6). Attribution, or more specifically, attribution style, refers to the way that individuals use to explain the causes of various life events, including positive and negative situations (7). When patients made negative instead of positive attributions to medical staff’s behaviors, they would show more aggression on medical staff. Thus, whether patients would have aggressive behavior against doctors is closely related to patients’ attributional style (8).

In fact, attributional style, as a special cognitive style, has already been demonstrated as an important individual factor affecting aggressive behavior (9). People would use internal factors or external factors to explain the causes of events. The former is called internal attribution and the latter is called external attribution (10–12). Fontao and Ross (13, 14) found that external attributional biases was associated to aggressive behaviors. The overuse of external attribution brought about higher levels of aggression. Cheng et al. (15) also discovered that attributional style could moderate the influence of frustration situation on aggression. In the doctor–patient relationship, hostility of patients would affect patients’ satisfaction for medical care and decrease their respect for physicians, bringing about more sharp conflicts between physicians and patients (16, 17). In this sense, it is vital to investigate the reasons why patients would produce attributional biases for physicians.

Patients were more likely to blame others and make attribution biases to the others for negative events than the healthy persons. When patients encountered the negative medical events, they may also attribute more to the physicians, which will cause more attributional biases against physicians (18–23). However, the previous studies concentrated more on the influence of patients related factors on their attributional biases against physicians, such as their illness type, symptom severity and personalities, failing to dig out physicians related factors that urge them to produce excessive external attribution and aggressive behaviors in medical situations (18, 19, 21, 22, 24).

It is worth noting that dehumanization is another key trigger for aggression toward others that can lead to violence in medicine (25, 26). Lekka et al. (27) showed that patients are dehumanized more mechanistically by medical professions than by the general population. Dehumanization in medical practice could generate deleterious consequences for patients, such as negative emotions, reduced self-esteem and relapse, which would influence patients’ perception for physicians and contribute to their attributional biases and aggressive behaviors when suffering negative medical situations (28, 29). Chiapperino and Boniolo (30) defined medical humanities as a humanistic problem-based approach to medicine on the purpose of combining “humane and humanizing” reflections on medicine with daily course of healthcare delivery. More specifically, from the perspective of doctor–patient relationship, the key elements of medical humanization included physician’s respect for patient’s dignity, uniqueness, individuality and humanity, empathy with patients, treating patients as persons instead of disease or symptoms, considering patients’ biopsychosocial and spiritual dimensions, respect for patient’s autonomy and patient involvement, verbal and non-verbal communication, etc. (31). In fact, in patients’ attributions, the main complaints and mismanagement of patients and their families involve their dissatisfaction with the abrasive, cold or callous attitudes of physicians or other health care providers. More accurately, they are dissatisfied with the dehumanized treatment they suffered (32). For example, Adams et al. (33) compared patients’ responses to dehumanizing doctors (indifferent to patients’ thoughts and feelings, treating patients as malfunctional machines, homogenization) and humanizing doctors (caring for patients’ thoughts and feelings, working together with patients to find personalized solutions that best suit their needs, person focused). They found that being treated by dehumanizing doctors, comparing with humanizing doctors, caused patients to feel dehumanized, reduce their satisfaction with doctors and decrease their expected compliance with treatment. Some studies also showed that physicians’ dehumanization towards patients in diagnosis and treatment (e.g., deindividuating practices, impaired patient agency, dissimilarity, mechanization, empathy reduction, and moral disengagement) will bring about patients’ dehumanization feelings toward the medical staff and the reduction of humanity attribution towards medical professions (29, 34, 35). With less humanity attribution to medical professions, patients are more likely to present attributional biases against medical workers, especially their attending physicians. On the contrary, humanization of care will increase patients’ medical satisfaction by responding to their needs, reduce fundamental attribution error, and further make patients establish proper internal and external attribution rather than attributional biases (36, 37). In this case, it is important for medical professionals to treat patients with humaneness and concern in healthcare so as to develop favorable patients’ attributions since medical humanization might be closely related to patients’ attributions, especially in negative situations.

Furthermore, it has been pointed out that more than half of the doctor–patient disputes and medical accidents handled by the Chinese Medical Association are caused by the lack of communication between doctors and patients (38). Doctor–patient communication is of great significance for the close doctor–patient relationship, promoting the satisfaction and rehabilitation of patients and reducing medical disputes (39, 40).

Patients’ evaluation for physicians in the communication is constructed by initial attribution (i.e., perceptions of their characteristics including ability, benevolence, and integrity) and deep attribution (i.e., locus of causality, controllability, and stability) (41, 42). By influencing patients’ initial attributions, the communication skills employed by physicians may lead to possible changes in patients’ deeper attributions. Inadequate or ineffective communication will lead to medication nonadherence and patients’ dissatisfaction (39). Patients with less adherence of treatment and medical satisfaction are more likely to attribute more responsibilities to their medical staff in medical malpractice cases (36, 43). Even so, the dilemma that doctor–patient relationship faces now is that doctors’ communication skills do not match patients’ needs (44), which might lead to more attributional biases by patients.

Nevertheless, prior research has also implied that communication has a close relationship with medical humanization (37, 45, 46). The humanization of health care is actually reflected in the communication, dialogue, relationship and interaction of medical professionals with patients and their families (46). Certain attitudes, behavior and skills, such as capacity to impart confidence, being empathetic, providing a ‘human touch’, relating on a personal level, being forthright, being respectful, and being thorough, are part of effective communication (45). If the main objective of medical humanization is to offer the best possible care and satisfy patients’ needs, communication with medical professionals committed to that objective is indispensable (37).

The influence of attribution style on patients’ cognition, emotion and behavior is the key to establish a harmonious and stable doctor–patient relationship and reduce doctor–patient conflicts or disputes, especially in negative medical events. Only by identifying the factors influencing patients’ attributional biases, it is possible for us to take reasonable and effective measures in medical education. However, few empirical studies have investigated their possible relationship. To make up the inadequacy, the present study would figure out the relationship among medical humanization perceived by patients in diagnosis and treatment, doctor–patient communication and the attributional style of patients in negative medical situations. It is hypothesized that both medical humanization and doctor–patient communication have positive predictive effects on the attributional style of patients and communication has the mediating effect on the other two variables. The finding might provide attainable intervention measures to promote more objective attributions of patients by improving doctors’ consciousness of humaneness and communication skills, and realize a harmonious doctor–patient relationship at the end.

## 2. Methods

### 2.1. Participants

A total of 3,000 participants were recruited by random sampling method from 103 general hospitals in the main cities of eastern, central and western regions of China during the period from September 2019 to February 2020. Meanwhile, the survey was conducted among various levels of hospitals involving primary, secondary, and tertiary hospitals. Participants were all patients who were older than 18 years and volunteered to take part in this survey. Since the survey adopted self-administered form, patients who were invited to participate in the survey after treatment could complete the questionnaires either by paper form or by electronic form at their convenience, but the survey data were collected by researchers confidentially. After the completion of data collection, we manually proofread all the collected data and removed the questionnaires whose response time was outside the plus or minus three standard deviations of the average response time. Thereby, a total of 2,256 valid questionnaires were obtained at the end.

### 2.2. Measures

Patients’ basic information, including gender, age, education, visiting hospital grade, and region, were collected at the beginning of the survey. And then patients were required to complete three questionnaires that were applied to measure the medical humanization perceived by patients in diagnosis and treatment, doctor–patient communication and attributional styles of patients in negative medical situations, respectively (Please see Supplementary material for the questionnaires here). Questionnaires for physicians’ humanization perceived by patients and attributional styles of patients in negative medical situations were self-designed by the researchers. The questionnaire for doctor–patient communication was a revised version of the Chinese version of SEGUE framework (C-SEGUE) for patients.

For the scale of medical staff’s humanization, it was designed for the group of patients to evaluate the humanness that physicians represented during their diagnosis and treatment, including the aspects of cognition, emotion, and behavior. 2 dimensions (Human Uniqueness and Human Nature) (47) and 10 items were involved in the scale with 5 positive items and 5 negative items totally. Questions in the dimension of Human Nature, such as “The medical staff are very human,” “Medical staff regard patients as mechanical cold robots. [reversed],” etc. are represented in the scale, which showed emotionality, agency, warmth and cognitive flexibility of humanity (47). Questions in the dimension of Human Uniqueness are also proposed, like “The medical staff stated the medical problems clearly and easily to understand.” to display the refinement, civility, morality and higher cognition of humanity (47). Five-point Likert scale was adopted for participants to assess the degree of approval on each item ranging from 1 (“Not at all”) to 5 (“completely agree”). The total score was 50 points with 5 marks for each item, in which the calculation of reversed questions was reversed. The higher the final score was, the greater humanization patients perceived. The Cronbach’s *α* of the humanity scale was 0.835, which indicated that the scale had good internal consistency. The scale also had good construct validity: RMSEA = 0.083, CFI = 0.987, TLI = 0.973, and CN = 415.

The SEGUE framework was developed by Northwestern University Medical School in North America (48) and the Chinese version was introduced by China Medical University in 2006 (49). There are 5 dimensions and 25 items, including “Set the stage,” “Elicit information,” “Give information,” “Understand the patient’s perspective” and “End the encounter.” Five-point Likert scale was applied to each item from “Never” to “All the time.” Higher score indicated better communication skills with patients. Since the target of the present study was patients rather than physicians, a little adjustment was made on the original C-SEGUE framework to make it more suitable for patients. For instance, the first item in the original questionnaire was “greet patient appropriately,” but it was revised as “greet me appropriately” in the current survey. The Cronbach’s *α* of the original C-SEGUE framework was 0.831 and the Cronbach’s *α* of the revised patient’s version was 0.970 in this study, which indicated that the scale had good internal consistency. The scale also had good construct validity: RMSEA = 0.061, CFI = 0.997, TLI = 0.993, and CN = 564.

The questionnaire for patients’ attributional style was self-designed and employed to measure the attributional style of patients for negative medical outcomes. This questionnaire was based on and adapted from a mature questionnaire that our research team designed before (50). The structure of the questionnaire was constructed and questionnaire items were compiled based on previous literature (10, 51, 52) and open-ended questionnaires for patients. Questions, like what are the negative situations/events that they often encounter in the hospital and what other events/scenes impressed them in the hospital, were set in the open-ended questionnaires. Delphi method was also applied to evaluate the applicability of the medical situations and concrete items. At the end, four events were obtained to reflect patients’ attribution in the four typical negative medical situations, respectively. Specifically, the four concrete negative medical situations in the scale included “The outcome is unsatisfactory after treatment,” “The doctor was impatient with me and had a perfunctory attitude,” “I felt very uncomfortable, but the doctor asked few questions and did not explain clearly” and “Finally it’s my turn, but the doctor was indifferent.” Concentrating on the internal versus external locus of causality, all the items were rated on the five-point Likert scale from 1 (“attributing to others or environmental factors”) to 5 (“Attributing to oneself”). It was noted that lower score represented higher degree of external attributional style. The Cronbach’s *α* of the patients’ attributional style questionnaire was 0.762, which indicated that the scale had good internal consistency. Meanwhile, the scale for patients’ attributional style had good construct validity: RMSEA = 0.035, CFI = 0.997, TLI = 0.987, and CN = 1,765.

### 2.3. Statistical analysis

SPSS Version 21.0 was used to conduct the data analysis, in which descriptive analysis, Pearson correlation and mediation analysis were processed. A model was built in order to examine whether doctors’ humanization view perceived by patients could influence doctor–patient communication and patients’ attributional style for negative medical events, and whether communication would play the mediating role on physicians’ humanization view perceived by patients and patients’ attributional style. All the continuous variables were standardized and the mean, standard deviations, and correlations of the variables were analyzed. Hayes’s Bootstrapping approach (53) was also applied to test the model proposed. Bootstrapping is not easily affected by the sample size and does not assume the normality of the mediation paths. Therefore, it can estimate the confidence interval more accurately. If 95% confidence interval (CI) does not contain the value zero, the mediation effect is significant.

## 3. Results

### 3.1. Participant characteristics

Totally 3,000 patients participated in the survey, of which 2,256 returned the valid questionnaires (returns-ratios 75.2%). The mean age of participants was 43.2 years old (SD = 15.9), among which 963 participants were male. The demographic characteristics of participants were shown in Table 1.

**Table 1:** Demographic characteristics of participants (*n* = 2,256).

Characteristics	*n*	(%)
Gender		
Male	963	42.7
Female	1,293	57.3
Age		
18–30	606	26.9
31–40	495	21.9
41–50	469	20.8
51–60	384	17.0
>60	302	13.4
Education background		
Primary school or below	253	11.2
Junior high school	534	23.7
Senior high school	506	22.4
Junior college	430	19.1
Undergraduate or above	533	23.6
Hospital grade		
Tertiary	1,778	78.8
Secondary	274	12.2
Primary	204	9.0
Region		
Eastern	683	30.3
Central	761	33.7
Western	812	36.0

### 3.2. Descriptive statistics and correlation analysis of the main variables

Based on the scale of patients’ attributions, the mean score was 8.24 (SD = 3.19) with the minimum value of 0 and the maximum value of 15. It was also found through further analysis on patients’ attributions that 56.9% of patients (*n* = 1,284) attributed the negative medical events or situations to the physicians completely. 12.5% of the patients (*n* = 282) had ambiguous attitudes and unclear attributions facing the negative medical events and situations. There were only 30.6% of patients (*n* = 690) who believed that the negative situations were not caused by physicians on purpose, and expressed that they cannot blame physicians wholly. Results showed that over half patients tended to make excessive external attributions. In addition, the average score given by the patients for medical staff’s humanization was 36.30 (SD was 5.80) with the minimum value of 10 and maximum value of 50. And the mean score for communication questionnaire was 101.11 (SD = 16.32) with the minimum value of 25 and maximum value of 125.

The mean scores, standard derivations and correlations of main variables were calculated shown in Table 2. Moreover, results indicated significant positive correlations between the three main variables (*r* = 0.112–0.236, *p* < 0.001), which represented that medical staff’s humanization was positively correlated with doctor–patient communication and patients’ attributional styles.

**Table 2:** Correlation analysis of main variables.

Characteristics	M	SD	Min	Max	1	2	3
1 Humanization of medical staff	36.30	5.80	13.00	50.00	1		
2 Doctor–patient communication	101.11	16.32	39.00	125.00	0.236***	1	
3 Attributions of patients	8.24	3.19	0.00	15.00	0.159***	0.112***	1

*N* = 2,256. ***p < 0.001.

### 3.3. Mediation analysis

The mediation effect of doctor–patient communication was tested by regression analysis and Bootstrapping approach. The results were shown in Table 3. When communication was the dependent variable, medical humanization perceived by the patients had significant effect on doctor–patient communication (M1). When patients’ attribution in negative medical situations was the dependent variable, medical staff’s humanization perceived by patients had significant predictive effect on the attributional style of patients in negative situations (M3). When patients’ attribution in negative situations was the dependent variable, and communication was put into the regression equation, it was found that both medical humanization and doctor–patient communication had significant predictive effects on the patients’ attributional styles in negative situation (M2). In general, doctor–patient communication was the mediator of the predictive effect of medical humanization on patients’ attribution in negative situations.

**Table 3:** Mediation analysis.

Predictors	M1: doctor–patient communication		M2: patients’ attribution in negative situations		M3: patients’ attribution in negative situations	
	*B*	*t*	*B*	*t*	*B*	*t*
Medical humanization	0.23**	11.52	0.14**	6.57	0.16**	7.64**
Doctor–patient communication			0.08**	3.71		
*R* ^2^	0.05		0.03		0.14	
*F*	132.76**		36.31**		30.56^**^	

*N* = 2,256. **p < 0.01.

**Figure 1:**
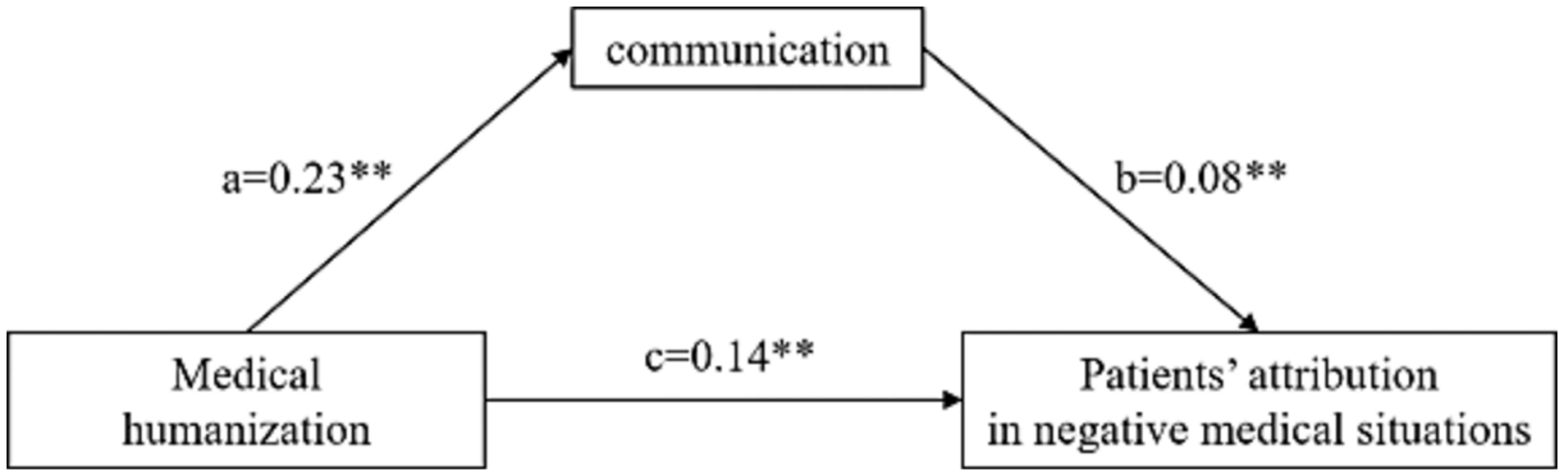
Mediation results of medical humanization on patients’ attribution in negative medical situations through communication. **p < 0.01. Direct path = c: *β* = 0.14, SE = 0.02, 95% CI [0.09–0.18]. The indirect path ab is a product of a path and b path. Indirect effect = ab: *β* = 0.02, SE = 0.01, 95% CI [0.02–0.03]. Total effect = ab + c: *β* = 0.16, SE = 0.09, 95% CI [0.48–0.85].

The model in Figure 1 displayed the mediation results of the relationship among medical humanization, doctor–patient communication and patients’ attribution in negative medical situations. Based on the model, two paths were presented in standard regression coefficients. It was found that medical humanization had both direct (*β* = 0.14, *p* < 0.01) and indirect (*β* = 0.02, *p* < 0.01) positive effects on patients’ attributional style in negative medical situations (*F* = 36.31, *r*^2^ = 0.03). The total standardized effect of medical humanization was strong (*β* = 0.16, *p* < 0.01). The mediation effect of communication occupied 12% of the total effect. Medical humanization was a stronger predictor of patients’ attributional style in negative situations than communication (*β* = 0.08, *p* < 0.01).

## 4. Discussion

It was found that both medical humanization and doctor–patient communication have effects on patients’ attribution in negative medical situations from their correlation analysis and the model above. To be more specific, medical staff’s humanization perceived by patients influences patients’ attributional style in negative medical events directly and indirectly through the communication between physicians and patients. The impact of medical humanization perceived by patients on patients’ attribution is slightly greater than that of doctor–patient communication on patients’ attribution in negative medical situations.

As expected, the predictive effect of medical humanization on patients’ attributional style was found in the present study, which could be explained by previous findings that attributions are usually based on personal relevance and experience (54). Hence patients normally assigned causality based on their medical experience. Since humanization embodied in the medical staff made patients have better personal and humanized experience during the medical encounter, patients will have more objective and kind attributions even in negative medical situations. The findings of this study are in line with previous research displaying that patients to be treated by physicians with humanistic philosophy have more feelings of humanization and are more satisfied with their physicians and treatment (33), which could lead to benign attribution of patients. Furthermore, the present study confirms the study results of Capozza et al. (55), illustrating the importance of humanity perception and humanity attributions in social relationships with patients.

On the other hand, the positive correlations between medical humanization and patients’ attribution also verifies the statement that dehumanization normally goes together with extreme situations marked by conflicts and violence (56). More precisely, dehumanization in medicine increased patients’ dehumanized feelings by arousing their negative emotions and deconstructing cognitive states, and eventually performed more external attributional biases and aggressive behaviors (26, 28, 57). The reason why patients in the present study made more external attributions facing negative medical situations could also be attributed to the dehumanized treatment by medical staff they felt in the hospitals. Bastian and Haslam (47) also demonstrated that people view themselves and others who ostracize them as less human when they have been socially excluded. In addition, previous studies have supported the importance of humanization of medical care for patients since patients themselves might exhibit aggressive behaviors (58, 59). Altogether, though dehumanization was demonstrated to be effective in reducing the symptoms of medical staff’s burnout (60), it has larger disadvantages in deceasing patients’ benign attribution, especially in negative medical situations, and further leading to medical violence.

Furthermore, consistent with our hypothesis, we found that communication has the effect on patients’ attributional style in negative medical situations, which could be partly explained by Tomlinson’s causal attribution model of trust repair (42). Black et al. (43) also found that patients who rely more on their physicians, trust their recommendations and question physicians less are less likely to blame their physicians when suffering medical errors. Thus, improving the adherence and trust of patients for physicians is one of the effective measures to promote the well-meaning and benign attribution of patients. Nevertheless, patients’ trust in physicians was based on their perception of physicians’ communication effort, which indicates that good communication is essential to establish trust between physicians and patients, and further promote the benign attribution of patients facing negative medical events (61). This finding also echoed with the study of Moore et al. (62). They found that positive physician communication behaviors made patients better perceive physicians’ abilities and reduced patients’ intention to claim negative medical outcomes from physicians and hospitals, therefore bringing about their benign attribution.

The present study also found that medical humanization has the influence on doctor–patient communication, which is consistent with the previous literature (46, 63, 64). For example, Basile et al. (65) found that dehumanization was perceived by patients in the “communication” when medical workers talked “over” patients and made distressing remarks in front of patients. Fontesse et al. (34) further put forward that dehumanization’s experience is anchored in negative social interactions. Poor patient physician communication decreases patient compliance to treatment strategies, patient satisfaction scores and on the extreme leads to violence directed to physicians (66). In humanized care, communication is an effective tool for establishing a good relationship between medical professionals and patients (46). To be humanized as medical staff and make patients perceive the medical humanization well call for effective communication (46).

All in all, this study is a real sense of initial attempt to demonstrate that medical humanity view perceived by patients affects their attributional style in negative medical situations through doctor–patient communication in empirical researches. The result reiterates the vital importance of medical staff’s humanization in promoting patients’ benign attributions in negative medical situations and establishing sound relationships with patients. In view of these, some measures should be developed to improve medical professions’ awareness about humanization. For instance, empathy is regarded as a requirement for overcoming dehumanization (67). Therefore, physicians’ empathy should be promoted by medical training. The requirement is also proposed to reduce the possibilities for healthcare workers to use dehumanization to alleviate professional exhaustion (34, 60). In addition, previous research raised some other methods to develop humanization in medicine (29). Taking an example, it is proposed that individuation should be promoted by making patients and medical professions more identifiable, such as by adding personalized details on patients’ and medical staff’s uniforms. Besides this, patients should be treated as active partners in clinical decision-making to enhance their empowerment and agency, and finally lower dehumanization. Ensuring that patients as well informed regarding their treatment and have an influence on the treatment would also attribute to control, agency and thus re-humanize them (68).

More importantly, developing interactions between patients and healthcare professionals may decrease dehumanization trend and further contribute to the benign attribution of patients (42, 69), which attaches great importance to the development of physicians’ communication skills. After all, communication is also critical in boosting patient satisfaction and compliance (39, 40). Therefore, it is quite necessary to implement effective communication skills training for medical professionals. Role-play, feedback and small group discussions are all illustrated as effective training strategies (70). It is also imperative for medical staff to know the issues of language and communication, such as Conversational Analysis. Ostermann and de Souza (71) have demonstrated the role of conversational analysis in doctor–patient communication. They also found that conversational analysis could be implemented to analyze how patients look upon concrete humanization practices in healthcare services and patients’ attributions. Besides, previous research discovered that the use of affiliation words, first-person singular pronouns, causation and differential words, and clout words will make patients perceive physicians’ compassionate care and respect to them (72).

Ultimately, given that most of researches focused on the superficial issues in medical education such as trainings for physicians’ communication skills (40, 73) and optimization of doctor–patient trust (33, 74), the present study deepened into more important themes of medical professional values such as medical humanization and training for patients’ attributions. The result of the study preliminarily expanded the vision of relevant research and improved the scope of medical research, proposing some implications for medical management, medical education and health education reform. For example, humanistic education for physicians should be included in healthcare management and medical education to make patients use rational judgment and objective attribution based on situational factors. Since medical staff’s humanization view is embodied in the process of communication with patients, specific modules on physicians’ humanistic treatment for patients and the corresponding communication skills training should be added in the syllabus for medical students and hospitals’ systematic management.

There are also some limitations in the current study. Firstly, the present study applies cross-sectional design, which only indicates that the variables we studied here have correlations with each other. But this kind of relationship might be causality or others. In view of this, interventional studies would be needed in the future studies to confirm whether this relationship is causal relationship. Secondly, there actually are more variables influencing patients’ attribution in negative medical situations, such as patients’ trust on physicians (42) and patients’ personality (43). But this study only focuses on the variables of humanity and communication. More variables could be explored in the future research. Finally, taking into account China’s national conditions and the first choice of hospitals for patients to be treated, this study was conducted mainly based on the patients in tertiary hospitals, with less proportion of patients in secondary and primary hospitals. However, it must be admitted that because of the better medical resources in tertiary hospitals, there are also more complex medical cases in tertiary hospitals, which might influence patients’ perceptions on doctors and hospitals. Thus, it should be paid more attention in the future research.

## 5. Conclusion

Patients’ attribution in negative medical situations is predicted by patients’ perception of medical staff’s humanization in healthcare and physicians’ communication skills directly and indirectly. Mediation analysis displays the mediator role of communication between medical humanization and patients’ attribution in negative medical situations. This study indicates that corresponding measures from the angles of medical humanization and doctor–patient communication should be taken to promote patients’ benign attribution in negative medical situations from different perspectives.

## Data availability statement

The raw data supporting the conclusions of this article will be made available by the authors, without undue reservation.

## Ethics statement

The studies involving human participants were reviewed and approved by the ethics committee of Shanghai Normal University. Written informed consent for participation was not required for this study in accordance with the national legislation and the institutional requirements.

## Author contributions

XC, PW, YW, QW, and FS conceptualized and designed the study. YW and QW collected the data. YW analysed the data. PW interpretated the data and drafted the manuscript. XC made contributions to the validation, guidance of revision, and supervision. All authors contributed to the article and approved the submitted version.

## Funding

This research was funded by Major bidding projects for National Social Sciences Fund of China, grant number 17ZDA327.

## Conflict of interest

The authors declare that the research was conducted in the absence of any commercial or financial relationships that could be construed as a potential conflict of interest.

## Publisher’s note

All claims expressed in this article are solely those of the authors and do not necessarily represent those of their affiliated organizations, or those of the publisher, the editors and the reviewers. Any product that may be evaluated in this article, or claim that may be made by its manufacturer, is not guaranteed or endorsed by the publisher.

## Supplementary material

The Supplementary material for this article can be found online at: https://www.frontiersin.org/articles/10.3389/fpubh.2023.1152381/full#supplementary-material
