# Supplementary material for: Influence of medical humanization on patients’ attribution in negative medical situations with communication as the mediator: a questionnaire study
Source: Front Public Health. 2023 Aug 31;11:1152381. doi: 10.3389/fpubh.2023.1152381 (PMC10501307; doi:10.3389/fpubh.2023.1152381)
Supplement: Supplementary file 1 [file Data_Sheet_1.doc]

Supplementary Material

# Influence of medical humanization on patients’ attribution in negative medical situations with communication as the mediator: a questionnaire study

Peijuan Wang, Yao Wang, Qing Wu, Fan Su and Xin Chang*

*** Correspondence:** Xin Chang, changxinwp@163.com

**Contents**

Appendix 1. Questionnaire for Physicians’ humanization perceived by patients

Appendix 2. Questionnaire for Attributional styles of patients in negative medical situations

Appendix 3. Questionnaire for Chinese version of SEGUE framework (C-SEGUE) for patients

**Appendix 1. Questionnaire for physicians’ humanization perceived by patients**

**Medical Humanization**

In terms of your past medical experience or what you know, do you think the following description is consistent with the performance of medical staff? Please read each item below carefully and choose the answer that you think is in line with the actual situation from the five options after each description.

| **Items** | **Not at all** | **Disagree** | **Not sure** | **Agree** | **Completely agree** |
| --- | --- | --- | --- | --- | --- |
| The medical staff are very human. | 1 | 2 | 3 | 4 | 5 |
| The medical staff state the medical problems clearly and easily to understand. | 1 | 2 | 3 | 4 | 5 |
| The medical staff respond patients’ needs in time. | 1 | 2 | 3 | 4 | 5 |
| The medical staff can self-discipline in the medical process. | 1 | 2 | 3 | 4 | 5 |
| The medical staff can handle medical problems rationally. | 1 | 2 | 3 | 4 | 5 |
| Medical staff believe that patients are cold. | 1 | 2 | 3 | 4 | 5 |
| Medical staff believe that the patients are emotionally numb. | 1 | 2 | 3 | 4 | 5 |
| Medical staff believe that in the face of medical problems, patients are easy to be impulsive. | 1 | 2 | 3 | 4 | 5 |
| Medical staff believe that in the face of medical problems, patients are simple-minded. | 1 | 2 | 3 | 4 | 5 |
| Medical staff believe that in the face of medical problems, patients are rude. | 1 | 2 | 3 | 4 | 5 |

**Appendix 2. Questionnaire for attributional styles of patients in negative medical situations**

***The complete questionnaire includes the attributional styles of patients in both positive (A, B, C, G) and negative medical situations (D, E, F, H). Here only the negative medical situations are listed in terms of the research purpose.**

**Patients’ Attribution in Negative Medical Situations**

The following questions are set up based on some virtual medical situations. Please imagine that if you encounter these events, please choose or write the answers that meet your actual situation.

**D. The outcome is unsatisfactory after treatment**

**Assuming this situation exists, you think the main reasons are:**

| **Items** | **Attributing to others or environmental factors** | |  | | | **Attributing to oneself** |
| --- | --- | --- | --- | --- | --- | --- |
|  | **1** | **2** | | **3** | **4** | **5** |
| 1. Do you think the reason why this happens is attributing to yourself, or to the others or environmental factors? |  |  | |  |  |  |
| 1. Would this happen again when you see the doctor or hospitalize next time? |  |  | |  |  |  |
| 1. Do the above reasons stated only affect your treatment or hospitalization, or all aspects of your life? |  |  | |  |  |  |
| 1. Can you control this reason? |  |  | |  |  |  |
| 1. Does the doctor do this on purpose? | Definitely not | Probably not | | Not sure | Probably yes | Definitely yes |
|  |  |  |  |  |  |  |
| 1. How angry do you feel about this situation? | Not at all | A little angry | | Angry | Quite angry | Absolutely furious |
|  |  |  |  |  |  |  |
| 1. How much do you want to blame the doctor? | Not at all | A little bit | | Half responsibility | Most responsibility | Whole responsibility |
|  |  |  |  |  |  |  |

**E. The doctor was impatient with me and had a perfunctory attitude**

**Assuming this situation exists, you think the main reasons are:**

| **Items** | **Attributing to others or environmental factors** | |  | | | **Attributing to oneself** |
| --- | --- | --- | --- | --- | --- | --- |
|  | **1** | **2** | | **3** | **4** | **5** |
| 1. Do you think the reason why this happens is attributing to yourself, or to the others or environmental factors? |  |  | |  |  |  |
| 1. Would this happen again when you see the doctor or hospitalize next time? |  |  | |  |  |  |
| 1. Do the above reasons stated only affect your treatment or hospitalization, or all aspects of your life? |  |  | |  |  |  |
| 1. Can you control this reason? |  |  | |  |  |  |
| 1. Does the doctor do this on purpose? | Definitely not | Probably not | | Not sure | Probably yes | Definitely yes |
|  |  |  |  |  |  |  |
| 1. How angry do you feel about this situation? | Not at all | A little angry | | Angry | Quite angry | Absolutely furious |
|  |  |  |  |  |  |  |
| 1. How much do you want to blame the doctor? | Not at all | A little bit | | Half responsibility | Most responsibility | Whole responsibility |
|  |  |  |  |  |  |  |

**F. I felt very uncomfortable, but the doctor asked few questions and didn't explain clearly**

**Assuming this situation exists, you think the main reasons are:**

| **Items** | **Attributing to others or environmental factors** | |  | | | **Attributing to oneself** |
| --- | --- | --- | --- | --- | --- | --- |
|  | **1** | **2** | | **3** | **4** | **5** |
| 1. Do you think the reason why this happens is attributing to yourself, or to the others or environmental factors? |  |  | |  |  |  |
| 1. Would this happen again when you see the doctor or hospitalize next time? |  |  | |  |  |  |
| 1. Do the above reasons stated only affect your treatment or hospitalization, or all aspects of your life? |  |  | |  |  |  |
| 1. Can you control this reason? |  |  | |  |  |  |
| 1. Does the doctor do this on purpose? | Definitely not | Probably not | | Not sure | Probably yes | Definitely yes |
|  |  |  |  |  |  |  |
| 1. How angry do you feel about this situation? | Not at all | A little angry | | Angry | Quite angry | Absolutely furious |
|  |  |  |  |  |  |  |
| 1. How much do you want to blame the doctor? | Not at all | A little bit | | Half responsibility | Most responsibility | Whole responsibility |
|  |  |  |  |  |  |  |

**H. Finally it’s my turn, but the doctor was indifferent**

**Assuming this situation exists, you think the main reasons are:**

**：**

| **Items** | **Attributing to others or environmental factors** | |  | | | **Attributing to oneself** |
| --- | --- | --- | --- | --- | --- | --- |
|  | **1** | **2** | | **3** | **4** | **5** |
| 1. Do you think the reason why this happens is attributing to yourself, or to the others or environmental factors? |  |  | |  |  |  |
| 1. Would this happen again when you see the doctor or hospitalize next time? |  |  | |  |  |  |
| 1. Do the above reasons stated only affect your treatment or hospitalization, or all aspects of your life? |  |  | |  |  |  |
| 1. Can you control this reason? |  |  | |  |  |  |
| 1. Does the doctor do this on purpose? | Definitely not | Probably not | | Not sure | Probably yes | Definitely yes |
|  |  |  |  |  |  |  |
| 1. How angry do you feel about this situation? | Not at all | A little angry | | Angry | Quite angry | Absolutely furious |
|  |  |  |  |  |  |  |
| 1. How much do you want to blame the doctor? | Not at all | A little bit | | Half responsibility | Most responsibility | Whole responsibility |
|  |  |  |  |  |  |  |

**Appendix 3. Questionnaire for Chinese version of SEGUE framework (C-SEGUE) for patients**

**SEGUE framework (C-SEGUE)**

Please choose the answer you think is in line with the actual situation from the five options after each description according to your actual medical experience.

| **Items** | **Never** | **Occasionally** | **Sometimes** | **Most of the time** | **All the time** |
| --- | --- | --- | --- | --- | --- |
| ***S*et the Stage** |  |  |  |  |  |
| **1. Greet me appropriately** |  |  |  |  |  |
| **2. Explain reasons for the inquiry to me (e.g.,** **assessing the clinical status, further diagnosis, and reporting to superior physician)** |  |  |  |  |  |
| **3.** **Introduce the agenda of consultation and physical examination (e.g., the issues and sequence of consultation, etc.)** |  |  |  |  |  |
| **4. Make a personal connection during visit (e.g., proper self-introduction, discussion beyond medical issues**) |  |  |  |  |  |
| **5. Maintain my privacy (e.g., knock, close door) to respect my rights of choice and privacy** |  |  |  |  |  |
| ***E*licit Information** |  |  |  |  |  |
| **6. Elicit my view of health problem and/or progress (ideas, concerns)** |  |  |  |  |  |
| **7. Explore physical/physiological factors (signs, symptoms)** |  |  |  |  |  |
| **8. Explore psychosocial/emotional factors (e.g., living situations, family relations, stress)** |  |  |  |  |  |
| **9. Discuss antecedent treatments with me (e.g., self-care, last visit, other care)** |  |  |  |  |  |
| **10. Discuss how health problem affects my life with me (e.g., quality-of-life)** |  |  |  |  |  |
| **11. Discuss lifestyle issues/prevention strategies with me (e.g., health risks)** |  |  |  |  |  |
| **12. Avoid asking me directive/leading questions** |  |  |  |  |  |
| **13. Give me opportunity/time to talk (e.g., don’t interrupt)** |  |  |  |  |  |
| **14. Listen. Give me undivided attention. (e.g., face me, verbal acknowledgement, nonverbal feedback)** |  |  |  |  |  |
| **15. Check/Clarify the information I gave (e.g., recap, ask “how much”)** |  |  |  |  |  |
| ***G*ive information** |  |  |  |  |  |
| **16. Explain rationale for diagnostic procedures to me (e.g., exam, tests)** |  |  |  |  |  |
| **17. Teach me about my own body & situation (e.g., provide feedback from exam/tests, explain anatomy/diagnosis)** |  |  |  |  |  |
| **18. Encourage me to ask questions** |  |  |  |  |  |
| **19. Adapt to my level of understanding (e.g., avoid/explain jargon)** |  |  |  |  |  |
| ***U*nderstand the Patients’ Perspective** |  |  |  |  |  |
| **20. Acknowledge my accomplishments/progress/ challenges (e.g., appreciation for my cooperation)** |  |  |  |  |  |
| **21. Acknowledge waiting time** |  |  |  |  |  |
| **22. Express caring, concern, empathy and make me feel warm and build confidence** |  |  |  |  |  |
| **23. Maintain a respectful tone to me** |  |  |  |  |  |
| ***E*nd the Encounter** |  |  |  |  |  |
| **24. Ask if there is anything else I would like to discuss** |  |  |  |  |  |
| **25. Review next steps with me** |  |  |  |  |  |
